# Augmentation mammaplasty by superolateral thoracic flap: a case report

**DOI:** 10.1186/s13256-021-03122-8

**Published:** 2021-11-17

**Authors:** Elise Lupon, Benoit Chaput, Thomas Meresse

**Affiliations:** 1 Service de Chirurgie Plastique et Reconstructrice, CHU Toulouse Rangueil, 1 Avenue Jean Poulhès, TSA 50032, 31059 Toulouse Cedex 9, France; 2 Department of Plastic and Reconstructive Surgery Research, Massachusetts General Hospital, 55 Blossom Street, Boston, MA 02114 USA; 3 Department of Plastic Surgery, Institut Universitaire du Cancer de Toulouse Oncopole, Institut Claudius Regaud, 1, Avenue Irène Joliot-Curie, 31059 Toulouse, France; 4 Department of Plastic surgery, University Toulouse III Paul Sabatier, Toulouse, France

**Keywords:** Bariatric surgery, Autologous breast augmentation, Fat compartment, Lateral chest wall, Superolateral thoracic flap

## Abstract

**Background:**

The lateral chest wall is intimately associated with the esthetics of the breast. Patients with massive weight loss often have excess skin and fat in the lateral thoracic region causing functional, esthetic, and psychological discomfort. In addition, the breasts exhibit extreme ptosis after weight loss due to a reduction in volume and projection that is exacerbated by qualitative changes in the skin, with loss of its natural elasticity. This article describes a reliable new technique for simultaneous autologous breast augmentation and lateral thoracic dermolipectomy to provide autologous tissue for breast augmentation and simultaneous rejuvenation of the chest wall.

**Case presentation:**

A 30-year-old Caucasian woman who had lost 58 kg after bariatric surgery had major skin excess sequelae combined with major breast ptosis. She wanted to correct her brachial and lateral thoracic skin and fat excess, as well as rejuvenate her breasts. The lateral thoracic panicle present was harvested and transposed in the retroglandular plane to perform autologous breast augmentation with lateral thoracic dermolipectomy.

**Results:**

The patient was totally healed and complication-free at day 15. Both esthetic results and patient satisfaction were good at 6 months post-surgery.

**Conclusions:**

Superolateral thoracic flap augmentation mammaplasty during thoracic dermolipectomy is a simple and safe procedure for selected patients. Durable and natural autologous breast augmentation may be achieved in a single step without the need for a breast implant, while rejuvenating the thoracic region.

## Introduction

One of the main issues in post-bariatric plastic surgery is to resect the dermolipomatous excess and to improve body contouring. Brachioplasty, abdominoplasty, cruroplasty, rhytidectomy, mammaplasty, and mastopexy are procedures that enhance self-esteem and reduce the health-related problems of these patients [1]. Ptosis and breast volume loss are common characteristics in women who have massively lost weight after bariatric procedures, and their correction is a frequent request [2].

Patients with massive weight loss also represent a group with distinct issues concerning the lateral chest wall [3]. The latter has the shape of a triangle with its apex at the axilla. It can be divided into three subunits: the axilla, breast, and chest wall. The lateral chest wall is intimately associated with the esthetics of the breast but is much neglected and rarely addressed as a separate unit [3]. Failure to address this area can have esthetic consequences and be a source of distress for patients, because the lateral chest wall and axilla cannot be easily hidden in certain clothing, such as bras and bathing suits.

In this article we describe a technique to address these issues. The lateral thoracic panicle juxtaposed to the breasts can easily be used as an adipofascial and superolateral thoracic (SLT) flap for breast augmentation. These SLT flaps on the lateral surface of the chest remain pedicled to their upper bloodstream and are buried subglandularly in the breast, creating an autologous breast augmentation.

We present the case of a young post-bariatric patient who received autologous breast augmentation with this lateral thoracic fat and skin excess at the same time as bilateral brachioplasty.

## Case presentation

### Patient and operative indication

A 30-year-old Caucasian woman consulted for surgical correction of excess brachial skin and fat and breast ptosis. According to the Regnault’s classification, she presented a grade II ptosis when she had her arms along her body. She wanted her breasts to be enlarged but without implants. She had undergone bypass bariatric surgery in 2015 for morbid obesity [body mass index (BMI) 51]. She had no major history other than a malabsorption syndrome following surgery. She was a nonsmoker. The loss of 58 kg had caused significant excess skin and fat. After stabilizing her weight (BMI 29), she underwent surgery to repair her abdominal and back weight loss sequelae 2 years after her bypass in 2017 thanks to a body lift.

On clinical examination, there was breast ptosis with a significant loss of projection and bilateral brachial excess skin, which is classic in this post-bariatric context. A major lateral thoracic excess causing significant discomfort was noted. Excess skin and subcutaneous tissue in the lateral chest wall was assessed by means of a pinch test to determine the available width of this donor flap. Considering these findings, we proposed brachioplasty associated with breast augmentation and intramammary burial of the excess tissue in the lateral thoracic region. The patient was informed of the expected higher degree of scarring. She was informed that the scars would extend from the inside of the elbow to the inside edge of the arm (brachioplasty), from the lateral surface of the thorax to the breast base (removal of excess lateral thoracic tissue and mammary burial), and that she would have a long submammary scar (necessary for positioning the flap).

**Fig. 1:**
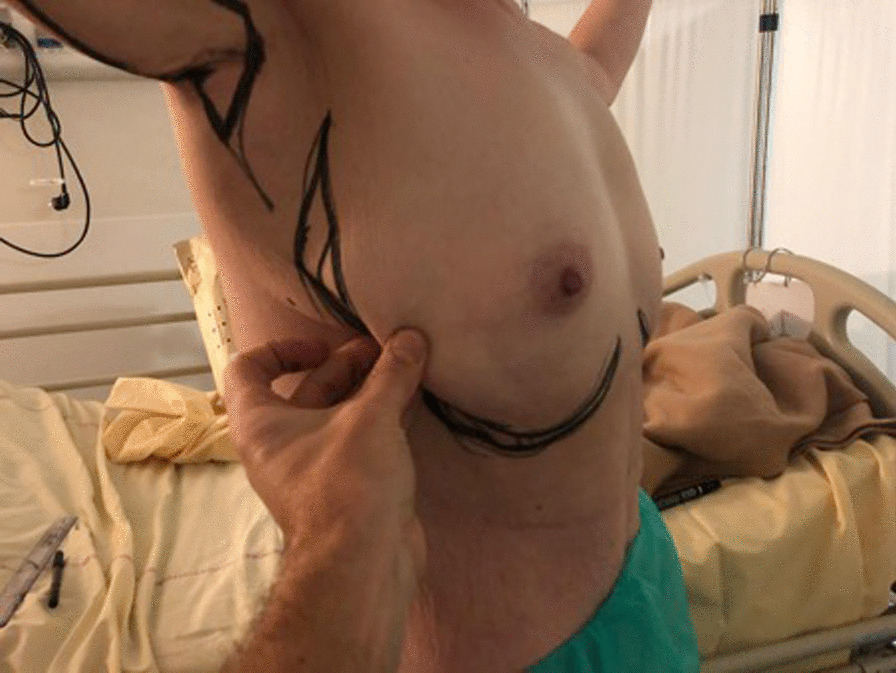
Reclination test of excess lateral fat skin and thoracic skin in breast

A maneuver that must be performed to convince both surgeon and patient of the eligibility for true SLT flap or augmentation mammaplasty by superolateral thoracic (SLT) flap is reclination of the lateral thoracic skin in the breast test (Fig. 1).

### Surgical technique

**Fig. 2:**
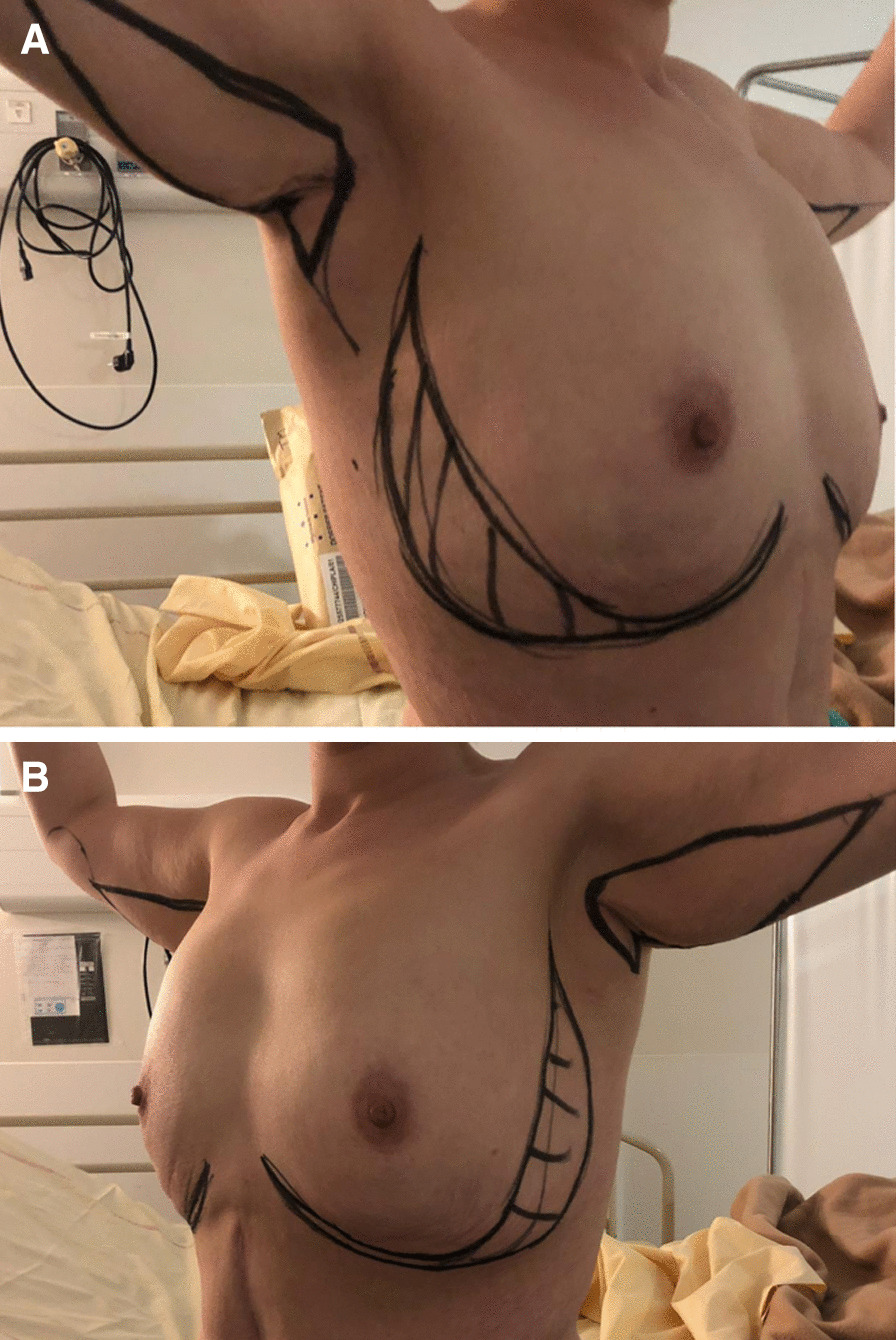
Preoperative drawing of excess skin and fat areas to be treated. Patient is standing. **A** Right view. **B** Left view

Preoperative drawing must be performed in a standing position (Fig. 2). Under general anesthesia, the patient is positioned supine on the operating table with both arms supported on arm boards abducted to 90°. The first step is classic brachial dermolipectomy. After a skin incision on the brachial drawing, de-epithelialization is performed with an electric scalpel. After hemostasis, closure is performed in two planes with 3/0 resorbable monofilament thread. This first step is carried out on both sides.

The second step consists of lateral thoracic skin resection, delimitation, and burial of the thoracic flaps. The shape of the resection must be confirmed by making a frame with staples. This frame allows the upper part of the design to be adapted and to create a continuity with the brachial dermolipectomy design with a cutaneous area preserved in an “S” shape between the two. This prevents a bridle when the arm is raised. The drawing is traced back to the frame, and the modeling staples can then be removed.

**Fig. 3:**
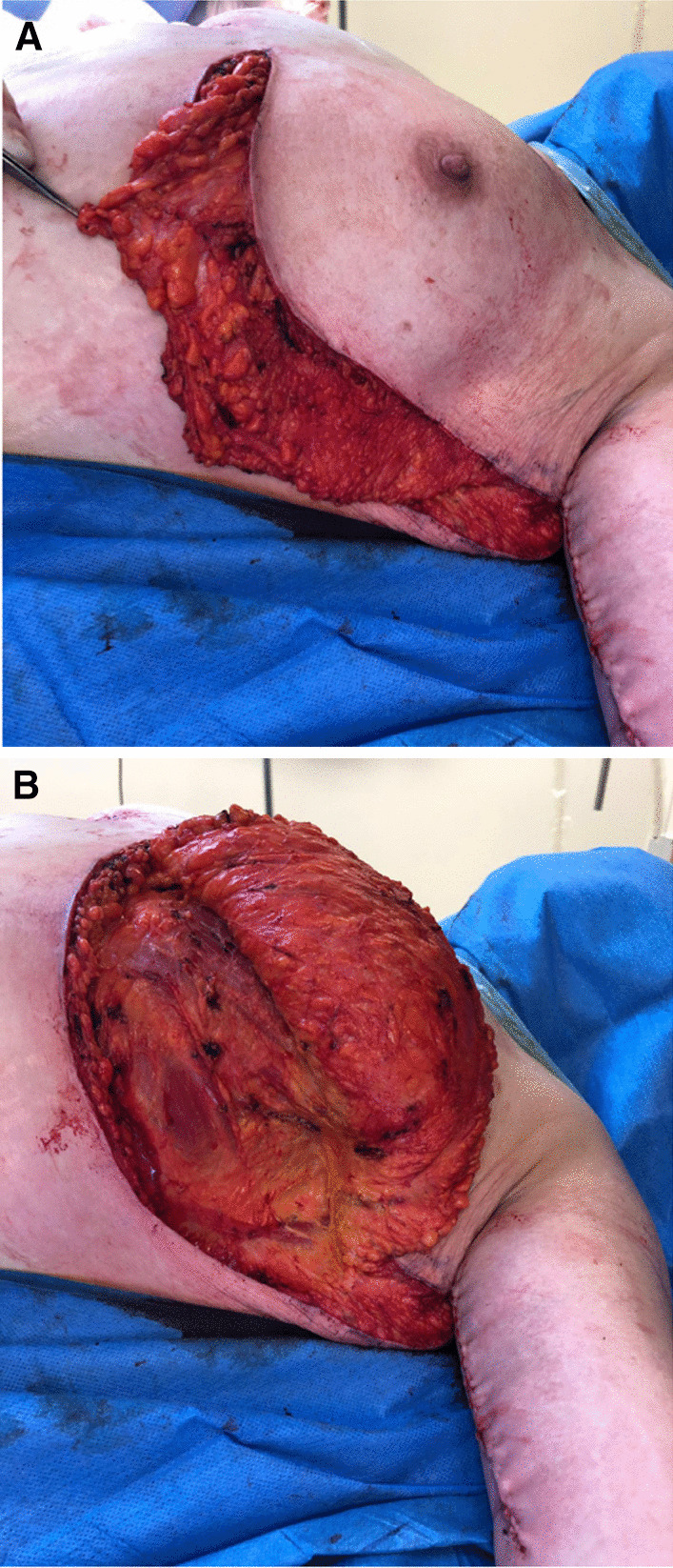
Detachment of the flap that is supplied by the external mammary vascular network. It is then attached to the edge on the breast side. **A** View of pedicle flap released from its distal attachments. **B** View of reclined pedicle flap

**Fig. 4:**
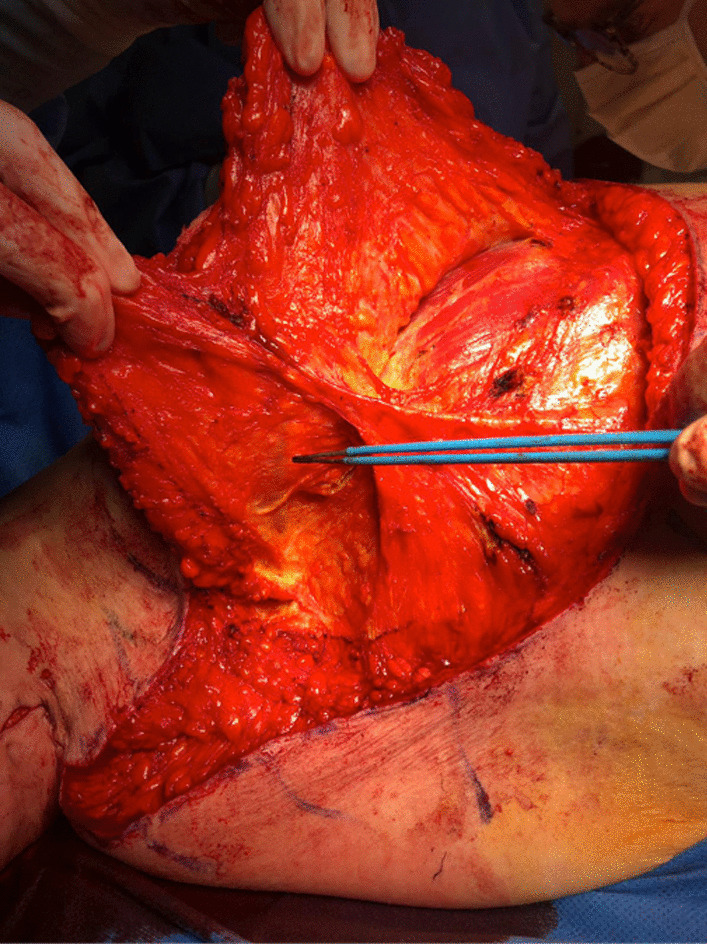
Visualization and conservation of external mammary vascular network

**Fig. 5:**
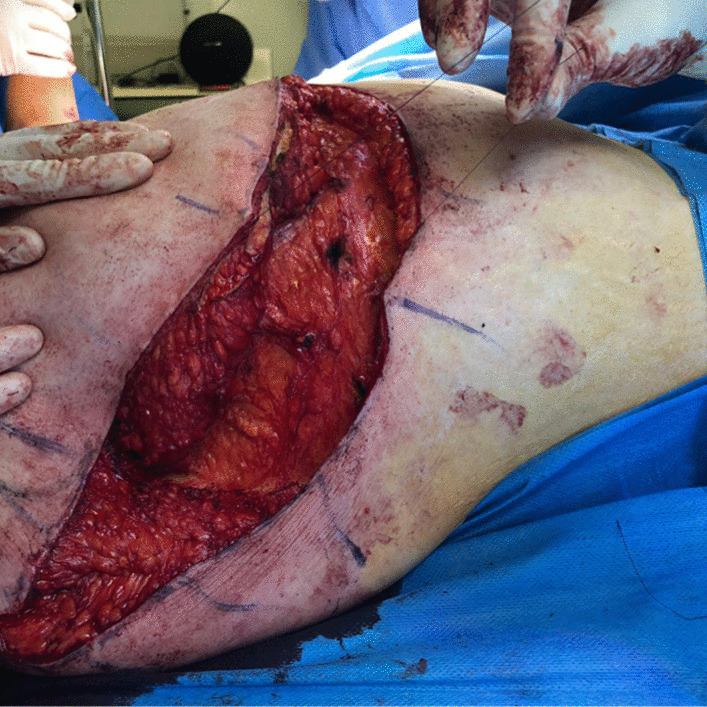
Burial of flap in breast and deep suturing

**Fig. 6:**
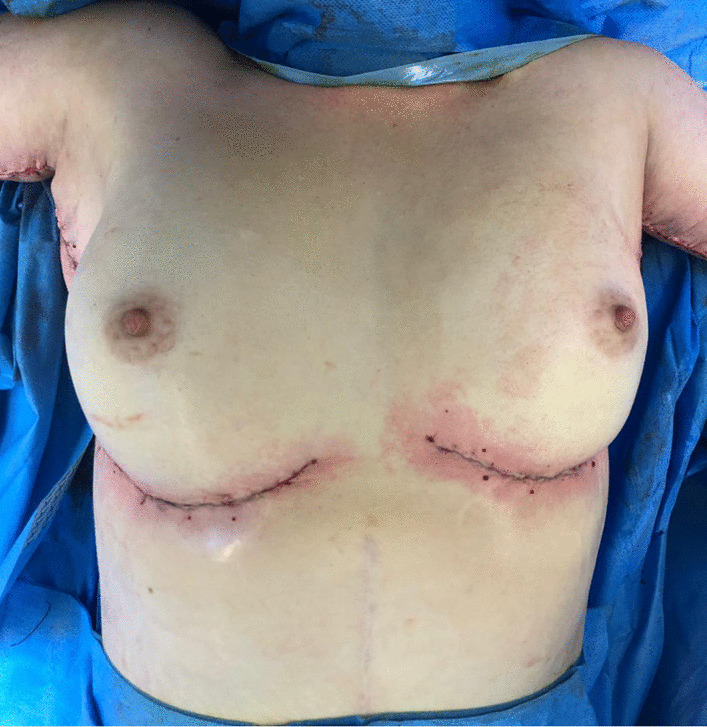
Result at end of procedure, symmetrical

De-epithelialization of the area is carried out with coagulating forceps. The flap is detached but remains attached to the bank on the breast side (Fig 3). During this maneuver, the external mammary vascular network must be preserved (Fig. 4). The flap is detached from the thoracic and pectoral planes to about half of the outer side of the breast. This flap is then buried into the breast and sutured deep down with 2/0 resorbable stitches (Fig. 5). Sutures are then made to reattach the submammary and external mammary groove, taking in the two edges and the periosteum of the corresponding rib with 2/0 resorbable stitches. This deep mooring is essential for the outcome to be satisfactory. Sutures are made between these pressure points with transparent resorbable 3/0 monofilament thread and an intradermal overlock with the same thread. This second step is carried out in the contralateral thoracic region. Care must be taken to ensure good symmetry at the end of the procedure (Fig. 6). No drainage system is required. To ensure better postoperative analgesia, all scars are infiltrated with ropivacaine.

### Postoperative care and results

**Fig. 7:**
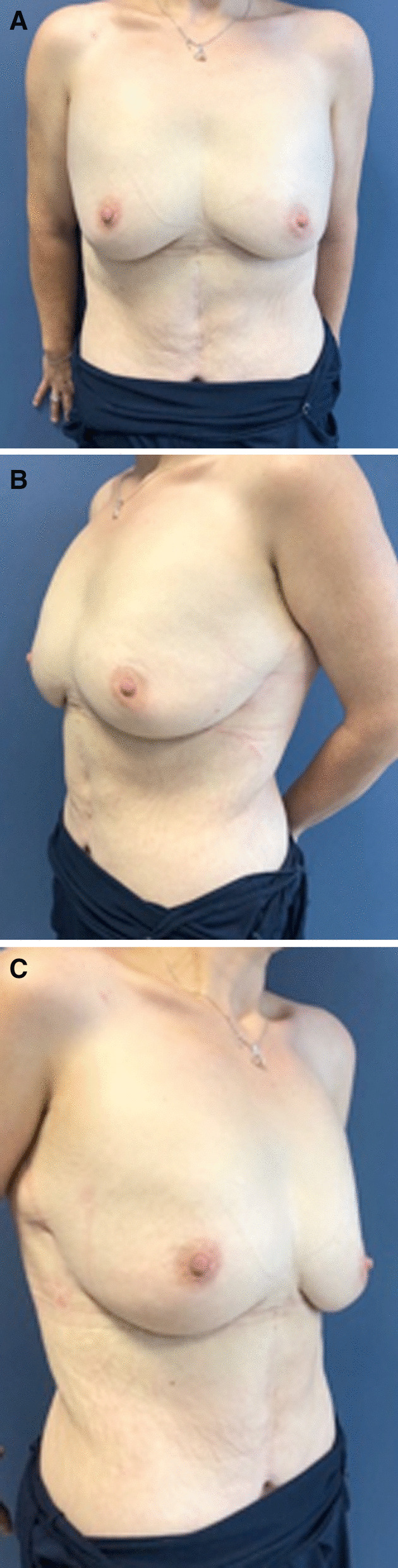
Result at 6 months. **A** Front view. **B** Left-side view. **C** Right-side view

The patient was hospitalized for 1 day and was allowed to leave the day after surgery. The postoperative course was uneventful, with no breast skin necrosis, no hematoma, and no seroma or infections. At 15 days, the patient did not have any scarring disorders and she was already satisfied with the result. At 6 months, she no longer had areolar ptosis with an areola above the fold under the breast when she had her arms along the body. Under the inframammary fold, a persistent slight glandular descent was noticeable but allowed to keep a natural aspect, according to the patient. She was very satisfied with the result and reported feeling much more comfortable from a functional and body image point of view (Fig. 7).

## Discussion

Plastic surgery after massive weight loss aims at resecting skin excess, facilitating personal hygiene to increase satisfaction with the body, improving sexual, social, and interpersonal relationships, increasing self-esteem, and providing better quality of life [4]. Patients with post-bariatric lateral chest wall deformities defined preoperatively and addressed directly have the greatest likelihood of obtaining a satisfactory esthetic outcome [3]. The augmentation mammaplasty technique by SLT flap described here allows the surgeon to perform symmetric augmentation of the breasts by using autologous tissue in patients with an unsightly excess of lateral thoracic skin and fat.

As the procedure is new, the patient stayed the first night after the operation in hospital in order to prevent any complications. However, given the low level of postoperative pain relieved by ropivacaine, the absence of expected complications with this type of surgery, and the lack of necessity to monitor the flap, outpatient hospitalization is probably sufficient.

Breast augmentation with or without mastopexy has regained its position as one of the most popular procedures in esthetic surgery. Various augmentation mastopexy techniques have been described to improve breast shape and increase their volume [5–7]. They are considered difficult to plan with results that are not easy to predict [8–10]. In our case, the placement of the breast flaps made it possible to reshape the breast sufficiently and to postpone classic mastopexy with its incumbent periareolar scars. However, our procedure cannot be performed at the same time as an inverted-T mastopexy in the event of major ptosis (Regnault stage IV). While augmentation with breast implants is still the simplest and safest method, complications such as capsular contracture, rippling, periprosthetic atrophy, implant deflation, and implant visibility and palpability may still occur. The use of autologous tissue for breast augmentation has several advantages over implants. Lateral thoracic tissue is often discarded, yet our technique allows it to be recycled for breast enhancement. There is no risk of capsular contracture or the other aforementioned problems, as the breasts are augmented with autologous tissue.

Breast augmentation with autologous tissue also creates a more natural feeling of having real breast tissue. Lipofilling can be performed subsequently if the volume of tissue is insufficient. SLT flap augmentation mammaplasty is not the only breast augmentation technique with autologous tissue. For example, mammaplasty by reverse abdominoplasty has been used successfully for rejuvenating the abdomen and the breast simultaneously [11–15]. In the augmentation mammaplasty by reverse abdominoplasty (AMBRA) technique [11], the upper abdominal panicle juxtaposed inferiorly to the breasts is poised for easy repositioning as adipofascial flaps to allow augmentation mammaplasty. Other autologous techniques exist such as bilateral de-epithelialized transverse rectus abdominis musculocutaneous flaps, extended transverse rectus abdominis musculocutaneous flaps, pedicled perforator flaps, and autologous fat transplantation, which are considered to have the same level of performance as synthetic implants [16–21].

Concerning the specific treatment of excess lateral thoracic fat and skin, few techniques have been reported to date. Some authors advocate an algorithmic approach to their surgical treatment, including transfer of autologous tissue, liposuction, fat injection, and direct excision. Cryolipolysis has also been used successfully to reduce discomfort from residual lateral chest wall fat in post-mastectomy patients [22]. The treatment of excess lateral thoracic fat and skin simultaneously with breast augmentation has been the subject of very few publications [23, 24].

Our technique is particularly adapted to E3 deformities in the classification of Bar-Meir *et al*. [3], involving an excess of both skin and fat of the lateral chest wall, usually involving all three subunits. Although initially designed for esthetic purposes, it has proven valuable for reconstructive purposes in appropriately selected cases [23]. Lateral thoracic tissue can be transferred to correct defects in treated or reconstructed breasts or to obtain symmetry. It has the advantage of combining autologous breast augmentation with an improvement in the lateral wall chest in a single procedure while keeping the groove under the breast. Its main theoretical risk is fat necrosis and cyst formation [25], hence the importance of preserving the external mammary vascular network for the vitality of the cutaneous sheath and breast fat, on the one hand, and of respecting the direct cutaneous perforators from the lateral thoracic, on the other. The flap is wide, which guarantees its vascular safety. The decision to lift the flap on a superior rather than an inferior external pedicle was considered carefully. This allows better anchoring of the scar and better fixation of the highly mobile fold under the breast in patients who have lost weight massively. This upper pedicle allows the flap to be turned over and folded to increase the projection of the breast.

Some authors [26] have demonstrated that the lateral thoracic region contains a fat compartment that is unique in its anatomical boundaries. Further vascularization of these lateral thoracic flaps has been described [24] and the pedicled fasciocutaneous flap can be based, as in this case, on direct cutaneous perforators from the lateral thoracic or intercostal arteries, or on septocutaneous perforators from the thoracodorsal or long thoracic arteries. According to the algorithm described by Levine *et al*. in 2015 [23] that facilitates the choice between the different pedicles, the flap must be centered over the fourth interspace if the tissue is needed for augmentation and if flap de-epithelialization is planned. Finally, the scar is less visible with this technique than with standard lateral thoracic dermolipectomy. Indeed, with lateral thoracic dermolipectomy, the vertical scar along the thorax is mandatory to remove any excess thoracic skin. Our technique allows the scars to be positioned advantageously on the external and submammary grooves, resulting in a much more esthetic appearance.

## Conclusions

SLT flap augmentation mammaplasty is a simple new procedure for patients with lateral thoracic skin laxity and adiposity who desire autologous tissue breast augmentation with or without mastopexy combined with rejuvenation of the lateral chest wall. The increased lateral flank scarring in continuity with a simultaneous brachioplasty incision is well tolerated by patients, with the additional benefit of reducing flank fullness and avoiding the more visible scars associated with mastopexy.

## Data Availability

Available (ask the corresponding author).

## Abbreviations

SLT: Superolateral thoracic flap
AMBRA: Augmentation mammaplasty by reverse abdominoplasty
